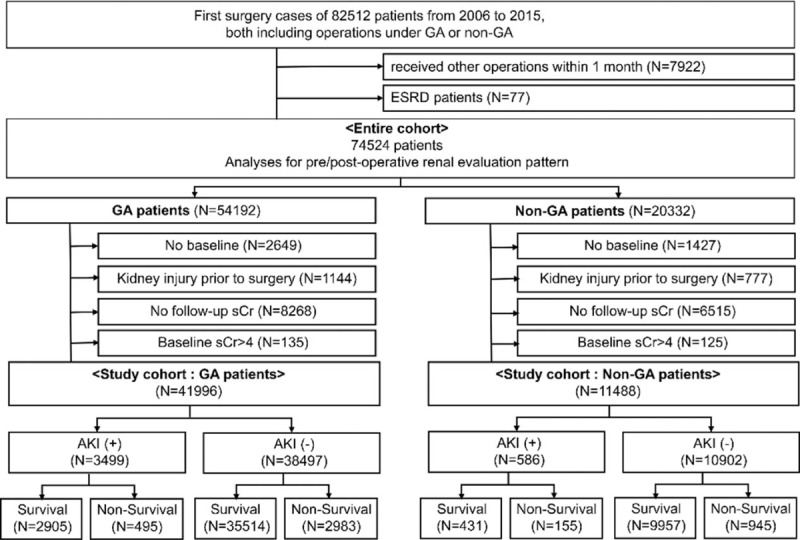# Awareness, incidence and clinical significance of acute kidney injury after non-general anesthesia: A retrospective cohort study: Erratum

**DOI:** 10.1097/MD.0000000000013399

**Published:** 2018-11-16

In the article, “Awareness, incidence and clinical significance of acute kidney injury after non-general anesthesia: A retrospective cohort study”,^[1]^ which appears in Volume 97, Issue 35 of *Medicine*, Figure 1 is incorrect and should be:

**Figure:**